# Alpha-chloralose poisoning in 25 cats: clinical picture and evaluation of treatment with intravenous lipid emulsion

**DOI:** 10.1177/1098612X241235776

**Published:** 2024-04-30

**Authors:** Sandra Lundgren, Kristoffer Dreimanis, Karolina Engdahl, Ulrika Windahl, Cecilia Tegner

**Affiliations:** 1 University Animal Hospital, Swedish University of Agricultural Sciences, Uppsala, Sweden; 2 AWAKE Animal Hospital, Stockholm, Sweden; 3 Evidensia (Uppsala Veterinary Clinic), Uppsala, Sweden; 4 Department of Clinical Sciences, Swedish University of Agricultural Sciences, Uppsala, Sweden; 5 Swedish National Veterinary Institute (SVA), Uppsala, Sweden

**Keywords:** Alpha-chloralose, poisoning, intoxication, Intralipid, intravenous lipid emulsion, rodenticide

## Abstract

**Objectives:**

The aims of this study were to describe the clinical picture and progression in cats with alpha-chloralose (AC) intoxication and to determine if treatment with intravenous (IV) lipid emulsion (ILE) influenced either the serum concentration of AC or the clinical signs.

**Methods:**

Cats with suspected AC poisoning admitted to a university small animal hospital were included. The cats were randomised into two groups: one receiving 20% ILE at a dose of 300 mg/kg as a 2 min bolus, followed by a 1500 mg/kg continuous rate infusion over 30 mins (IL+ group) and the other receiving IV fluid therapy with Ringer’s acetate (IL− group). Serum samples were drawn at 0, 2, 12 and 24 h after admission. Samples were tested for AC with a novel validated, quantitative, ultra-high-performance liquid chromatography-tandem mass spectrometry method. Vital and predefined clinical signs were noted at the times of sampling and patients were scored using a previously described intoxication severity score. Telephone interviews were conducted after discharge to assess outcome.

**Results:**

A total of 25 cats were enrolled: 13 cats in the IL+ group and 12 in the IL− group. The most common clinical signs at presentation were tremor (n = 22, 88.0%), cranial nerve deficits (n = 20, 80.0%) and bradycardia (n = 19, 76.0%). No significant difference in AC concentration or change in intoxication score over time was found between the IL+ and IL− groups at any time point (*P* >0.05). All cats recovered within 72 h.

**Conclusions and relevance:**

ILE did not have any effect on the AC serum concentration or clinical signs in AC-poisoned cats. All cats survived until follow-up. In cats with an acute onset of the described neurological signs, AC intoxication is an important differential diagnosis with an excellent prognosis.

## Introduction

Alpha-chloralose (AC) was introduced as a painless alternative to other rodenticides and avicides.^1^ After ingestion, AC is metabolised to chloral and trichloroethanol, which exert dose-dependent excitatory and anaesthetic effects on the central nervous system in low and high doses, respectively, causing severe bradycardia, hypothermia, coma and death in high doses.^2.3^ Poisoned cats often experience a second excitatory phase before returning to normal, if they do not succumb during the depressive phase.^3^ Sensitivity to AC is species-specific, and cats are particularly vulnerable.^2^ Publications on accidental AC poisoning in cats are available.^4–8^ The median lethal dose in early studies was reported as 400–600 mg/kg PO for cats and dogs by Hanriot and Richet (1893, as cited by Balis and Monroe).^3^ The minimum lethal dose PO is 100 mg/kg for cats, compared with 600–1000 mg/kg for dogs.^1^ AC is used for anaesthesia in experimental studies, with reported anaesthetic doses for cats in the range of 25–100 mg/kg chloralose intravenously as bolus doses^9–11^ or 5 mg/kg/h as a constant rate infusion (CRI).^12^ Even though some cats might ingest AC bait directly,^8^ they are generally considered dietary neophobes and are reluctant to ingest bait.^1^ A multicentre study concluded that secondary poisoning (relay toxicosis), where a cat ingests intoxicated prey, was most common.^5^

In AC-poisoned cats, ataxia, hyperaesthesia, tremor, cranial nerve deficits (mainly visual impairment, miosis, mydriasis and reduced pupillary light reflex [PLR]) and behavioural abnormalities are commonly seen (see Videos 1–5 in the supplementary material).^4–6,8^ Seizures, coma and potentially lethal hypothermia may be seen after ingestion of high toxic doses, with reported mortalities in the range of 0–18%.^4–8^ As AC has no known antidote, treatment is supportive with decontamination whenever possible, reducing sensory stimuli to limit hyperexcitability, thermal support and anticonvulsants, if needed.

Intravenous lipid emulsions (ILEs) are used as lipid resuscitation in life-threatening local anaesthetic toxicity in humans.^13–16^ They have also been reported as adjunctive therapy for intoxications with benzodiazepines, tricyclic antidepressants, anticonvulsants, beta blockers and calcium channel blockers.^16^ The mechanism of action of ILEs in detoxification is incompletely understood. Proposed effects include lipid partitioning – where the effect of toxic lipophilic solutes is limited through redistributing the substances from their site of action–mitochondrial recovery enhancement and direct cardiac inotropy.^16–20^

Adjunctive treatment with ILEs in carprofen, bupivacaine, lidocaine, metaldehyde and ivermectin intoxications in cats has been reported.^21–26^ One prospective, randomised study described ILE treatment in permethrin-intoxicated cats.^27^ Although seemingly well tolerated, anaphylactic reactions and persistent hypertriglyceridemia with corneal lipidosis after ILE infusions in cats have been reported.^28,29^ There are also concerns that treatment with ILEs might worsen intoxication via the increased uptake of certain lipophilic drugs.^30^ Anecdotal use of Intralipid (IL) (Fresenius Kabi, 200 mg/ml) has been reported as successful as an off-label therapy in AC poisoning in cats,^4^ but no published study has investigated a possible effect.

The objectives of this study were to describe the clinical picture and progression in cats with confirmed AC poisoning and clinical signs of intoxication, and to determine if treatment with IL influenced either the serum concentration of AC or the clinical signs.

## Materials and methods

The study was approved by the Uppsala Animal Experiment Ethics Board, Sweden (reference number 5.8.18-10303/2020). Owners gave written consent with the possibility of withdrawing their cats from the study at any point.

This prospective, randomised clinical study was conducted at the Small Animal Clinic at the Swedish University Animal Hospital (UDS) in Uppsala, Sweden between January 2020 and February 2022. Cats admitted to the emergency department with a suspicion of AC poisoning were eligible for participation.

The inclusion criteria were as follows: outdoor cats with possible exposure to AC and at least two of the predefined clinical signs (see Table 1 in the supplementary material).^4^ Indoor cats without access to AC and cats with signs of concurrent diseases or that were negative for AC were excluded.

The cats were randomised into two groups: the first receiving intravenous (IV) IL (treatment group; IL+) and the second receiving IV maintenance fluid therapy with Ringer’s acetate (Fresenius Kabi) only (control group; IL–). Randomisation was performed using an online simple randomisation plan generator (www.randomization.com) that randomised numbers 1–50 as IL+ or IL–. For both groups, blood samples were drawn at 0, 2, 12 and 24 h after admission. The test tubes were centrifuged at 12,300 × *g* for 5 mins (VMR Microstar 12), and serum was separated and transferred into Eppendorf tubes and stored at –20°C. All samples were analysed for AC at the Swedish Veterinary Institute (SVA) using a novel validated, quantitative, ultra-high-performance liquid chromatography-tandem mass spectrometry method.^31^

After the first sampling, cats in the IL+ group were treated with IL IV at a dose of 300 mg/kg (1.5 ml/kg) as a 2 min bolus, followed by a 1500 mg/kg (7.5 ml/kg) CRI over 30 mins. This dose has previously been recommended in acute neurological and cardiological toxicity with lipophilic substances.^19^ Additional supportive treatments and diagnostic tests were at the discretion of the attending veterinarian.

Clinical personnel assessed vital signs and noted clinical signs as present or absent in a standardised form (see Table 2 in the supplementary material) at the time of each sampling. In addition, all cats were scored using a previously described intoxication severity score (see Table 3 in the supplementary material),^4^ where scores of 1–2 represent ambulatory cats with milder and/or fewer clinical signs and scores of 3–4 represent non-ambulatory cats with more severe signs of intoxication.

The duration of hospitalisation was noted, and telephone interviews were conducted 10–20 days after discharge for follow-up, where the owner/carer was asked about recovery.

The statistical analyses were performed using R version 4.0.0 (R Foundation). Continuous variables were presented as median (min–max) and categorical variables as number and percentage per category. The Shapiro–Wilk test was used to evaluate the normal distribution of continuous variables. Comparisons of sex and neuter status, body weight and age between the IL+ and IL− groups were performed using Fisher’s exact test, Student’s *t*-test and Wilcoxon’s rank sum test, respectively. AC concentration in the IL+ and IL− groups and changes in AC concentration over time were compared between groups using Wilcoxon’s rank sum test. The intoxication severity scores in the IL+ and IL− groups and differences in scores between the sampling time points were compared using Fisher’s exact test. *P* values <0.05 were considered statistically significant.

## Results

A total of 27 cats with suspected AC poisoning were enrolled. All cats presented between September and January, except two that presented in March (2021) and August (2020), respectively. Two cats were excluded: one at the owner’s request and one that was negative for AC, leaving 13 cats remaining in the IL+ group and 12 in the IL− group.

Of the 25 cats in the study, 18 (72.0%) were domestic shorthairs, six (24.0%) were domestic longhairs and one (4.0%) was a crossbreed (Persian/Maine Coon). Population demographics are summarised in Table 1. There was a significant difference in sex distribution between the IL+ and IL− groups, with 50.0% (n = 6) males in the IL− group and 92.3% (n = 12) males in the IL+ group (*P* = 0.030). No significant difference in age or body weight was found (*P* = 0.327 and *P* = 0.569, respectively).

**Table 1:** Demographic information concerning sex, neuter status, body weight and age of 25 cats with alpha-chloralose poisoning

	IL+	IL−	Total
Sex and neuter status			
Female, entire	1 (7.7)	4 (33.3)	5 (20.0)
Female, spayed	0 (0)	2 (16.7)	2 (8.0)
Male, entire	1 (7.7)	0 (0)	1 (4.0)
Male, castrated	11 (84.6)	6 (50.0)	17 (68.0)
Body weight (kg)	4.6 (1.25–6.2)	4.35 (1.8–5.5)	4.6 (1.25–6.2)
Age (years)	4.0 (0.25–20.5)	2.5 (0.42–7.5)	3.50 (0.25–20.5)

Data are n (%) or median (range)

IL = Intralipid (Fresenius Kabi, 200 mg/ml)

The most common clinical signs at presentation were tremor (n = 22, 88.0%), cranial nerve deficits (n = 20, 80.0%) and bradycardia (n = 19, 76.0%). The cranial nerve deficits most frequently seen were visual impairment (n = 11, 44.0%), miosis (n = 9, 36.0%), mydriasis (n = 2, 8.0%) and reduced PLR (n = 11, 44.0%). As well as the signs recorded in the standardised form, one cat also had periods with a third-degree atrioventricular block, which resolved spontaneously. Clinical signs and their progression over time are described in Table 2.

**Table 2:** Clinical signs and their progression over time in 25 cats with confirmed alpha-chloralose poisoning

	Sample 1 (0 h)		Sample 2 (2 h)		Sample 3 (12 h)		Sample 4 (24 h)	
Clinical signs	IL+	IL−	IL+	IL−	IL+	IL−	IL+	IL−
Tremor	12 (92.3)	10 (83.3)	11 (84.6)	6 (50.0)	3 (23.1)	2 (16.7)	0 (0)	2 (16.7)
Cranial nerve deficits	10 (76.9)	10 (83.3)	11 (84.6)	8 (66.7)	5 (38.5)	4 (33.3)	0 (0)	2 (16.7)
Bradycardia	9 (69.2)	10 (83.3)	8 (61.5)	9 (75.0)	6 (46.2)	7 (58.3)	4 (30.8)	4 (33.3)
Hyperaesthesia	7 (53.8)	6 (50.0)	5 (38.5)	7 (58.3)	3 (23.1)	5 (41.7)	1 (7.7)	1 (8.3)
Non-ambulatory	3 (23.1)	8 (66.7)	3 (23.1)	9 (75.0)	3 (23.1)	4 (33.3)	0 (0)	0 (0)
Hypothermia	5 (38.5)	6 (50.0)	2 (15.4)	3 (25.0)	2 (15.4)	1 (8.3)	1 (7.7)	0 (0)
Behavioural changes	6 (46.2)	5 (41.7)	7 (53.8)	3 (25.0)	6 (46.2)	8 (66.7)	5 (38.5)	5 (41.7)
Ataxia	7 (53.8)	3 (25.0)	5 (38.5)	0 (0)	0 (0)	0 (0)	0 (0)	0 (0)
Somnolence	4 (30.8)	6 (50.0)	5 (38.5)	4 (33.3)	3 (23.1)	2 (16.7)	0 (0)	3 (25.0)
Stupor	2 (15.4)	3 (25.0)	2 (15.4)	4 (33.3)	1 (7.7)	2 (16.7)	0 (0)	0 (0)
Hypotension	1 (7.7)	2 (16.7)	1 (7.7)	2 (16.7)	1 (7.7)	2 (16.7)	0 (0)	0 (0)
Coma	1 (7.7)	1 (8.3)	1 (7.7)	1 (8.3)	0 (0)	0 (0)	0 (0)	0 (0)
Seizures	2 (15.4)	0 (0)	0 (0)	1 (8.3)	0 (0)	0 (0)	0 (0)	0 (0)
Bradypnoea	1 (7.7)	0 (0)	0 (0)	1 (8.3)	1 (7.7)	0 (0)	0 (0)	0 (0)
Hyperthermia	0 (0)	0 (0)	0 (0)	0 (0)	1 (7.7)	0 (0)	0 (0)	0 (0)

Data are n (%)

IL = Intralipid (Fresenius Kabi, 200 mg/ml)

**Figure 1:**
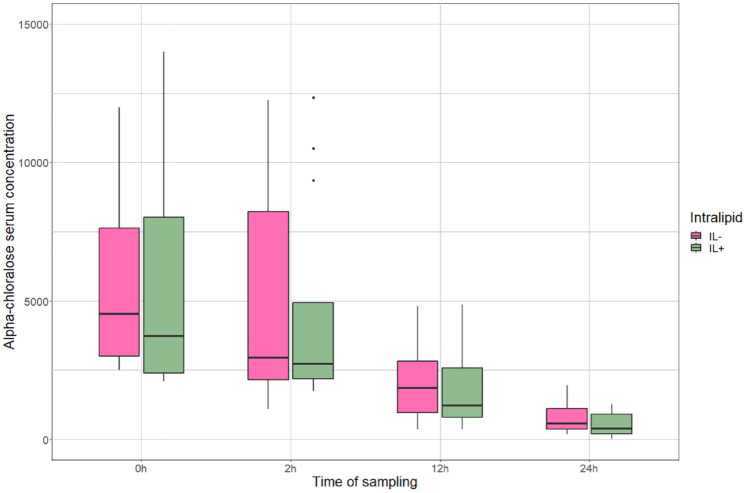
Change in serum AC concentration in the treatment (IL+) and control (IL–) groups over time, presented as AC concentration in ng/ml. The box displays the median and IQR and the whiskers (vertical bars) IQR*1.5. The points represent outliers. AC = alpha-chloralose; IL = Intralipid (Fresenius Kabi, 200 mg/ml); IQR = interquartile range

In total, 90 serum samples were available for analysis and the total AC concentrations were in the range of 15–14,000 ng/ml: 15–14,000 ng/ml in the IL+ group and 176–12,250 ng/ml in the IL− group. No significant difference in AC concentration was found between the IL+ and IL− groups at any time point. The decrease in AC concentration over time at 0–2 h (*P* = 0.389), 2–12 h (*P* = 0.668), 12–24 h (*P* = 0.270) and 0–24 h (*P* = 0.348) was not significantly different between the groups (Figure 1).

Intoxication severity scores are presented in Table 3. The severity scores differed significantly between the groups at 0 h and 2 h (*P* = 0.007 and *P* = 0.009, respectively). The change in score over time was not significantly different between the groups at 0–2 h (*P* = 0.582), 2–12 h (*P* = 1.000) and 12–24 h (*P* = 0.155). Three cats (12.0%, n = 1 IL+, n = 2 IL–) had an increase in severity score at 0–2 h, one of which (group IL–) also had an increase in AC serum concentration. In addition, one cat (group IL–) had an increase in AC serum concentration at 0–2 h with an unchanged severity score.

**Table 3:** Intoxication severity scores over time in 25 cats with confirmed alpha-chloralose poisoning

Time	IL+	IL−	Total	Missing scores	*P* value*
0 h	2 (1–4)	3 (1–4)	2.5 (1–4)	3	**0.007**
2 h	2 (1–3)	3 (1–4)	2 (1–4)	3	**0.009**
12 h	1 (1–3)	3 (1–3)	1.5 (1–3)	7	0.510
24 h	1 (1–1)	1 (1–1)	1 (1–1)	10	–

Data are median (range). Significant P values are in bold

* Comparing the scores of the IL+ and IL− groups

IL = Intralipid (Fresenius Kabi, 200 mg/ml)

In 22/25 cats, additional blood analyses were performed (see Table 4 in the supplementary material). One cat had mild azotaemia (creatinine 224 µmol/l; reference interval [RI] 53–141) at presentation, which had resolved when tested the next day; one cat had a mild to moderate increase in alanine aminotransferase (ALT 6.5 µkat/l; RI 0–2), where the owner declined follow-up; two cats had mild hypoglycaemia (3.3 mmol/l and 3.8 mmol/l, respectively; RI 3.9–6.7); five cats had mild hypokalaemia (2.6–3.0 mmol/l; RI 3.1–4.0); and three cats had mild hyperkalaemia (4.2–4.4 mmol/l).

Supportive treatment with IV Ringer’s acetate (Fresenius Kabi) was provided for all cats. A heating pad (3M Bair Hugger) and/or a blanket was used for hypothermic cats (n = 11, 44.0%); and eye lubrication and oxygen support was provided for stuporous or comatose cats (n = 7, 28.0%). Two cats (8.0%) with hypokalaemia received IV potassium chloride and two cats (8.0%) with mild hypoglycaemia received IV glucose as a bolus and as a 2.5% CRI, respectively. Five cats (20.0%) were treated with benzodiazepines against seizures or seizure-like activity and one cat (4.0%) received an additional single IV bolus of phenobarbital because it was refractory to benzodiazepines. One cat (4.0%) was treated with maropitant. All cats were initially monitored in the intensive care unit before being transferred to the medicine ward when considered clinically stable, usually the day after admission.

In total, 21 cats (84.0%) were discharged from the hospital with minor or no remaining clinical signs within 24 h of admittance. The remaining four cats (16.0%), two from the IL+ group and two from the IL− group, were discharged within 48 h. At follow-up, the owners of two cats (8.0%) reported transient haematochezia shortly after the cats arrived home. All cats recovered completely within 72 h, and none died or were euthanased.

## Discussion

IL treatment of AC-poisoned cats showed no effect compared with the controls; no significant differences were found between the groups with regard to clinical intoxication severity score, serum concentration of AC over time, duration of hospitalisation or outcome.

The possible therapeutic effect of ILE treatment in AC-poisoned cats was evaluated in this study as there are several anecdotal reports of its positive effects in intoxicated cats, where the cats have been described as rapidly recovering when ILE was infused. This phenomenon is likely attributable to the characteristics of the intoxication; when AC wears off, some cats may wake up quite abruptly, which, by coincidence, could occur when ILE treatment is administered to the individual cat. On the other hand, treatment with ILE did demonstrate clinical efficacy in a retrospective study^32^ of a wide range of poisonings, including AC, showing the need for prospective, controlled and randomised veterinary studies.

The mechanism of action of ILE is not fully understood. The most common theory is the lipid partitioning theory where ILE exerts its effect by redistributing lipid-soluble substances from their site of action.^16–20^ AC has both hydrophobic and hydrophilic properties, which supported the idea of a therapeutic effect from ILE infusion in AC-poisoned cats. Since no effect was recorded, AC is either not lipophilic enough, with a log*P*_O/W_ (o/w = octanol/water) of 1.020 where a log*P* <1 indicates hydrophilicity and >1 lipophilicity, or is unaffected by IL due to other factors.

Although no positive effects were recorded from ILE treatment in AC-poisoned cats, neither were any adverse effects noted. A recent study reviewing the effects of ILE on amitriptyline in a rat model found an increased blood concentration of amitriptyline in the treated rats, due to the enhancement of drug absorption from the gastrointestinal tract.^30^ The results of the present study showed no such tendency, as the serum concentration of AC did not increase in the treated cats. IL is a relatively safe drug to use in toxicities, but more studies are needed regarding its mechanism of action and potential use.

This study shows that the prognosis for cats with the degree of exposure and intake of AC described in the current study was excellent with supportive treatment, in concordance with previous publications.^4,5^ In the present study, all cats recovered completely from signs of intoxication within 72 h. However, there have been reports of more guarded prognoses. In publications by Segev et al^6^ and Dijkman et al,^8^ the mortality rate was 15% and 18%, respectively. Compared with those cohorts, the cats in the present study presented with milder clinical signs, with only one cat (7.7%) presenting comatose compared with almost 50% in earlier studies. The most frequently noted clinical signs on presentation in the current study were tremor (n = 22, 88.0%), cranial nerve deficits (n = 20, 80.0%) and bradycardia (n = 19, 76.0%). In a retrospective study by Tegner et al,^4^ the most common clinical signs were similar, with ataxia, tremor and cranial nerve deficits being most prevalent. The reason for differences in severity could be due to varying ingested doses or the point in the intoxication progression when the cats are brought to the clinic. However, since the study by Segev et al^6^ did not include a quantitative analysis of blood concentrations of AC and the study by Dijkman et al^8^ did not confirm poisoning by including any toxicological analyses, no certain comparisons can be made between studies regarding either ingested dose or clinical presentation.

Severe AC poisoning causes hypothermia, which can be lethal, and concurrent bradycardia, bradypnoea and hypotension worsen the prognosis without adequate supportive treatment. It can be speculated that the cats in our study with only minor clinical signs would have survived without hospitalisation. However, two cats showed increases in serum AC concentration between 0 and 2 h and three cats showed increased intoxication severity scores during the same time. The ingested dose of AC for any outdoor cat will be unknown, meaning that the potential for deterioration will be impossible to predict at presentation, especially since there may be ongoing uptake in the gastrointestinal tract. However, most cases in the present study declined rapidly once clinical signs had developed, either at home or after admission, and we recommend monitoring all cats with AC poisoning for at least a few hours, even cats with minor clinical signs.

AC may cause seizures in severe intoxications, and five of the cats in the present study were treated with anticonvulsants against seizures or seizure-like activity. However, in our experience, most cases do not suffer from true seizures, but from hyperaesthesia with associated twitches or myoclonus due to an increased sensitivity to sensory stimuli. This seems to be most evident in the excitatory phase, when the comatose or stuporous cat is starting to regain normal consciousness. In most cases, it is sufficient to keep the cat in a quiet environment with minimal handling and the hyperaesthesia will wear off. The effect of benzodiazepines in AC-poisoned cats was not evaluated in the present study, but in our experience, treatment with benzodiazepines might prolong the recovery time. Further studies would be needed to objectively evaluate the use of anticonvulsants in the treatment of seizures or seizure-like activity in AC-poisoned cats.

There was a statistically significant difference in sex distribution between the groups, with more males in the IL+ group. Overall, there were more male cats than female cats in this study. As there was no difference in clinical severity score or serum AC concentration between groups, this potential bias is considered negligible. This could be a coincidence due to the small sample size, but it can also be speculated that male cats might hunt more, or that there are differences in hunting patterns between male and female cats regarding preferences for hunting healthy, active or slow, poisoned mice.

Aside from AC analysis, all haematological and biochemical testing was performed based on a case-by-case assessment by the attending clinician. There were minor abnormalities, which in a few cases (n = 4, 16.0%) led to a change in treatment. Two cats were treated for hypokalaemia with IV potassium supplementation. Two cats were treated for hypoglycaemia, of which one also had seizure-like activity. However, the hypoglycaemia was not deemed to be the cause of the seizure-like activity but rather a result, as the hypoglycaemia was mild. In both cases, the hypoglycaemia resolved after glucose supplementation and was normal at follow-up a few hours later. With such a minor abnormality, an analytical error must also be considered. One cat had mild azotaemia that was assessed as prerenal and resolved with fluid therapy. One cat had a mild to moderate elevation of ALT that was considered incidental as it was the only case with this finding. The other abnormalities were considered clinically insignificant and the treatment was not changed.

Shortly before the start of the study, the use of AC by members of the general public was banned in Sweden, leading to a drastic decrease in the number of AC cases admitted to UDS. Thus, the sample size may have been too small to show an effect of ILE treatment (type II error). Further, due to human error, 10 AC serum samples were unfortunately missing at the time of analysis.

## Conclusions

Treatment with IL 20% IV infusion had no effect on serum AC concentration or the progression of clinical signs over time compared with supportive treatment. Supportive treatment remains the treatment of choice in feline AC poisoning and the prognosis for cats with the level of AC poisoning recorded in the present study is excellent.

## Supplemental Material

Table 1: Clinical definitions.

Table 2: Standardised form.

Table 3: Intoxication severity score.

Table 4: Additional blood analyses.

Supplementary Material Descriptions_cat_videos.
