# Supplementary material for: Alpha-chloralose poisoning in 25 cats: clinical picture and evaluation of treatment with intravenous lipid emulsion
Source: J Feline Med Surg. 2024 Apr 30;26(4):1098612X241235776. doi: 10.1177/1098612X241235776 (PMC11103310; doi:10.1177/1098612X241235776)
Supplement: Table 1: [file sj-docx-1-jfm-10.1177_1098612X241235776.docx]

Supplementary Table 1. Clinical definitions

**Coma** - non-ambulatory, obtunded, unconscious and unresponsive including to nociceptive stimuli.

**Stupor** - non-ambulatory, obtunded, unresponsive to external stimuli except nociceptive stimuli. May have some degree of consciousness.

**Ambulatory** - ability to rise and take at least 10 weight bearing, unassisted steps without falling.

**Somnolence** - impaired reactions to external stimuli and reduced awareness.

**Seizures** - tonic/clonic epileptiform seizures in an unconscious patient.

**Cranial nerve affection** – including, but not limited to: miosis, mydriasis, anisocoria, impairment of pupil light reflex, dazzle or menace, facial nerve paresis, vestibular signs, ptyalism.

**Vision impairment** - signs indicating reduced/impaired ability to process visual stimuli. These patients are also noted as “cranial nerve affection”.

**Ataxia** - decreased ability to coordinate movements, including proprioceptive, cerebellar and vestibular ataxia.

**Hypotension** - average systolic pressure ≤100 mmHg or mean arterial pressure ≤80 mmHg.

**Hypothermia** - body temperature ≤37^o^C.

**Bradycardia** - heart rate ≤140/min.

**Bradypnea** - respiratory rate ≤15/min.

**Other respiratory alteration** - choppy breathing pattern, paradoxical breathing, intermittent apnoea or other abnormal breathing patterns.

**Hyperesthesia** - exaggerated reactions to external sensory stimuli such as sound, light or touch.

**Tremor** - rhythmic oscillating movement generalised or localised, especially involving ears, whiskers and muzzle.

**Behavioural changes** - in patients with normal consciousness. Includes signs of hallucinations (subjective), extreme polyphagia, disorientation, euphoria, dysphoria, hypervigilance, compulsive behaviour or aggression in a patient who is normally calm/timid
