# Supplementary material for: Alpha-chloralose poisoning in 25 cats: clinical picture and evaluation of treatment with intravenous lipid emulsion
Source: J Feline Med Surg. 2024 Apr 30;26(4):1098612X241235776. doi: 10.1177/1098612X241235776 (PMC11103310; doi:10.1177/1098612X241235776)
Supplement: Table 2: [file sj-docx-2-jfm-10.1177_1098612X241235776.docx]

| ***Circle present clinical signs*** | **Sample** | **Mental status** | **Movement** | **Clinical parameters** | **Cranial nerves** | **Behaviour** |
| --- | --- | --- | --- | --- | --- | --- |
| ***Patient ID, place label*** | Sample 1 (0h)  Time and date:  ILE given before sampling?  *Yes No*  Medication given before sampling?  *Yes No*  If yes, what? | Normal  Somnolence  Stupor  Coma | Ambulatory  Non-ambulatory  Tremor  Ataxia  Hyperesthesia  Seizures | Temperature:  HR:  RR:  Blood pressure:  Abnormal breathing pattern?  *Yes No* | Pupils:  *Normal  Miosis  Mydriasis*  *Anisocoria*  PLR:  *Normal  Abnormal*  Visual impairment:  *Yes No*  Salivation  *Yes No* | Aggression  Polyphagia  Hallucination/disorientation  Anxiety  Other, specify:  Other comments to clinical signs: |
|  | Sample 2 (2h)  Time and date:  ILE given before sampling?  *Yes No*  Medication given before sampling?  *Yes No*  If yes, what? | Normal  Somnolence  Stupor  Coma | Ambulatory  Non-ambulatory  Tremor  Ataxia  Hyperesthesia  Seizures | Temperature:  HR:  RR:  Blood pressure:  Abnormal breathing pattern?  *Yes No* | Pupils:  *Normal  Miosis  Mydriasis*  *Anisocoria*  PLR:  *Normal  Abnormal*  Visual impairment:  *Yes No*  Salivating  *Yes No* | Aggression  Polyphagia  Hallucination/disorientation  Anxiety  Other, specify:  Other comments to clinical signs: |
|  | Sample 3 (12h)  Time and date:  ILE given before sampling?  *Yes No*  Medication given before sampling?  *Yes No*  If yes, what? | Normal  Somnolence  Stupor  Coma | Ambulatory  Non-ambulatory  Tremor  Ataxia  Hyperesthesia  Seizures | Temperature:  HR:  RR:  Blood pressure:  Abnormal breathing pattern?  *Yes No* | Pupils:  *Normal  Miosis  Mydriasis*  *Anisocoria*  PLR:  *Normal  Abnormal*  Visual impairment:  *Yes No*  Salivating  *Yes No* | Aggression  Polyphagia  Hallucination/disorientation  Anxiety  Other, specify:  Other comments to clinical signs: |
|  | Sample 4 (24h)  Time and date:  ILE given before sampling?  *Yes No*  Medication given before sampling?  *Yes No*  If yes, what? | Normal  Somnolence  Stupor  Coma | Ambulatory  Non-ambulatory  Tremor  Ataxia  Hyperesthesia  Seizures | Temperature:  HR:  RR:  Blood pressure:  Abnormal breathing pattern?  *Yes No* | Pupils:  *Normal  Miosis  Mydriasis*  *Anisocoria*  PLR:  *Normal  Abnormal*  Visual impairment:  *Yes No*  Salivating  *Yes No* | Aggression  Polyphagia  Hallucination/disorientation  Anxiety  Other, specify:  Other comments to clinical signs: |

**Supplementary Table 2. Standardised form. Clinical signs at the time of sampling** **in 25 cats with confirmed alpha-chloralose poisoning**

*ILE:* intravenous lipid emulsion, *HR:* heart rate, *RR:* respiratory rate, *PLR:* pupillary light reflex
