# Supplementary material for: Alpha-chloralose poisoning in 25 cats: clinical picture and evaluation of treatment with intravenous lipid emulsion
Source: J Feline Med Surg. 2024 Apr 30;26(4):1098612X241235776. doi: 10.1177/1098612X241235776 (PMC11103310; doi:10.1177/1098612X241235776)
Supplement: Table 3: [file sj-docx-3-jfm-10.1177_1098612X241235776.docx]

**Supplementary Table 3. Intoxication Severity Score**

| **Clinical signs** | **Score** | **Definition** |
| --- | --- | --- |
| ***Ambulatory*** | | |
| Mild | 1 | Minor and/or fewer than four clinical signs |
| Moderate | 2 | Moderate and/or more than three clinical signs |
| ***Non-ambulatory*** | | |
| Severe | 3 | Severe and more than five clinical signs except those mentioned for score 4 |
| Very severe | 4 | Sever and more than five clinical signs, including coma, seizures, and apnoea. Also including patients without coma, seizures or apnoea, but with severe affection of at least two of the following: systolic blood pressure (<80 mmHg), heart rate (<80bpm), respiratory rate (<10 bpm), or body temperature (<35^o^C) |
