# Supplementary material for: Alpha-chloralose poisoning in 25 cats: clinical picture and evaluation of treatment with intravenous lipid emulsion
Source: J Feline Med Surg. 2024 Apr 30;26(4):1098612X241235776. doi: 10.1177/1098612X241235776 (PMC11103310; doi:10.1177/1098612X241235776)
Supplement: Table 4: [file sj-docx-4-jfm-10.1177_1098612X241235776.docx]

**Supplementary Table 4. Additional blood analyses in 25 cats with confirmed alpha-chloralose poisoning**

| Analysis | n | RI | Number of cats with abnormal results | Abnormal values |
| --- | --- | --- | --- | --- |
| ABL90 Flex Plus (Radiometer Medical ApS) | | | | |
| pH | 22 | 7.29–7.45 | 0 |  |
| HCO3^-^ (mmol/L) | 22 | 17.2–24.4 | 4 | 25.7 |
|  |  |  |  | 26.4 |
|  |  |  |  | 26.6 |
|  |  |  |  | 30.6 |
| pvCO2 (mmHg) | 22 | 29.0–47.7 | 2 | 48.7 |
|  |  |  |  | 49.5 |
| Na^+^ (mmol/L) | 22 | 150–161 | 1 | 163 |
| K^+^ (mmol/L) | 22 | 3.1–4.0 | 8 | 2.6 |
|  |  |  |  | 2.7 |
|  |  |  |  | 3.0 |
|  |  |  |  | 3.0 |
|  |  |  |  | 3.0 |
|  |  |  |  | 4.2 |
|  |  |  |  | 4.2 |
|  |  |  |  | 4.4 |
| Cl^-^ (mmol/L) | 22 | 115–129 | 3 | 110 |
|  |  |  |  | 112 |
|  |  |  |  | 114 |
| iCa^2+^ (mmol/L) | 22 | 1.20–1.40 | 2 | 1,16 |
|  |  |  |  | 1,41 |
| Glucose (mmol/L) | 22 | 3.9–6.7 | 5 | 3,3 |
|  |  |  |  | 3.8 |
|  |  |  |  | 7.6 |
|  |  |  |  | 7.6 |
|  |  |  |  | 8.3 |
| Lactate (mmol/L) | 22 | <2 | 2 | 2.3 |
|  |  |  |  | 3.3 |
| Hct (%) | 22 | 29–55 | 0 |  |
| ProCyte Dx (IDEXX Laboratories, Inc) | | | | |
| WBCs (x10^9^/L) | 7 | 2.87–17.02 | 0 |  |
| Hct (%) | 7 | 30.3–52.3 | 0 |  |
| ADVIA 2120i (Siemens Healthineers) | | | | |
| WBCs (K/µL) | 3 | 4.8–19 | 0 |  |
| Hct (%) | 3 | 29–50 | 1 | 28 |
| Catalyst Dx (IDEXX Laboratories, Inc) | | | | |
| Creatinine (µmol/L) | 10 | 53–141 | 1 | 224^1^ |
| ALT (U/L) | 10 | 12–130 | 0 |  |
| ALP (U/L) | 8 | 14–192 | 0 |  |
| Total protein (g/L) | 6 | 57–89 | 0 |  |
| Albumin (g/L) | 6 | 22–40 | 0 |  |
| ARCHITECT c4000 (Abbott) | | | | |
| Creatinine (µmol/L) | 6 | 70–160 | 0 |  |
| ALT (µkat/L) | 6 | 0–2 | 1 | 6.5 |
| ALP (µkat/L) | 3 | 0–1 | 0 |  |
| Total protein (g/L) | 3 | 62–84 | 1 | 54 |
| Albumin (g/L) | 5 | 23–34 | 1 | 21 |
| SAA (mg/L) | 5 | <10 | 0 |  |

^1)^ Follow-up sample next day, analysed at ARCHITECT c4000 normalised, creatinine 155 µmol/L (RI 70-160 µmol/L)

RI = reference interval; HCO3^-^ = bicarbonate; pvCO2 = partial pressure of carbon dioxide in venous blood; Na^+^ = sodium; K^+^ = potassium; Cl^-^ = Chloride; iCa^2+^ = ionized calcium; Hct = haematocrit; WBCs = white blood cells; ALT = alanine aminotransferase; ALP = alkaline phosphatase; SAA = serum amyloid A
