# Supplementary material for: Alpha-chloralose poisoning in 25 cats: clinical picture and evaluation of treatment with intravenous lipid emulsion
Source: J Feline Med Surg. 2024 Apr 30;26(4):1098612X241235776. doi: 10.1177/1098612X241235776 (PMC11103310; doi:10.1177/1098612X241235776)
Supplement: Supplementary Material [file sj-docx-5-jfm-10.1177_1098612X241235776.docx]

Video 1

Ambulatory cat (not seen on this video) with severity score 2. Showing normal interaction with surroundings, but evident ataxia and tremors. Typical signs in moderate intoxication or in early or recovery phases of more severe intoxications.

Video 2

Non-ambulatory - severity score 3 - cat showing classical signs with abnormal mentation, twitches, and tremor. These signs may be seen both in early intoxication and during the recovery phase. Note the cotton in the cat’s ears to decrease auditory stimulus that may worsen the signs.

Video 3

Non-ambulatory cat with myoclonus and severity score 3. Note the lack of cotton in the ears and the increased myoclonus coinciding with auditory stimuli in the background.

Video 4

Cat with severity score 3, displaying evidence of somnolence with abnormal response to stimuli and no signs of interaction. This cat is at this stage not showing any of the described excitatory signs such as tremors, twitches or hyperesthesia.

Video 5

Comatose cat with severity score 4.
